# Trephining for Apoplexy

**Published:** 1898-06-04

**Authors:** 


### TREPHINING FOR APOPLEXY.

A case is reported in the New York *Medical Record*, in which Dr. R. T. Morris opened the skull in a case of ingravescent apoplexy, with the object of lessening the amount of injury to the brain which was likely to be produced by the pressure of the effused blood. The case terminated fatally, but was full of interest on the one hand from the very considerable relief which immediately followed the operation, and on the other from the fact that the localising symptoms did not show the locality of the effusion, so that while the case emphasises the possible utility of the operation, if the seat of the hæmorrhage can be found, it clearly shows that one must not be led to operate by the apparent definiteness with which the signs point to the locality of the effusion. The case was that of a man fifty-six years of age, who while walking across the room felt that his right arm was becoming paralysed. After five minutes his right leg was paralysed, and at the end of thirty minutes he was unconscious and vomiting. Dr Morris saw the patient about forty-five minutes after these symptoms had developed. Having decided to operate, he opened the skull over the ascending parietal convolution, *i.e.*, over the arm centre, because the arm was first involved. At this time the patient was so nearly moribund that the operation was for a moment discontinued. On the meninges being opened, however, a very large quantity of pink serum rapidly escaped, spurting out under great pressure. The man's breathing immediately became regular, and his pulse dropped nearly to the normal and remained so. The exploration was carried into the lateral ventricle, but very little serum was found there. It was then extended towards the island of Reil, and some small clots escaped from the wound in the brain made by the instruments. Here the patient rallied as one would from a clot in the brain. By the second day he was able to sit up and take some food. The effect of the operation was to relieve the general pressure, and thus to prevent immediate dissolution, although no clot was found. The patient, however, developed a hypostatic pneumonia on the tenth day, and died on the twelfth, having in the meantime remained hemiplegic on the right side—arm, leg, and face. He was also aphasic in every sense—word-deafness, word-blindness, and inability to express a single idea. He knew the use, however, of objects presented to him. The autopsy showed an enormous clot in the fissure of Sylvius leading inward, and in the anterior portion of the brain.

It appeared that in this case the sensory aphasia was not due to the direct influence of the clot, but to the interference with the circulation in the sylvian artery cutting off the supply of blood to the part of the brain in which the centres affected were situated. In commenting on this case Dr. Weir mentioned that in 1894 he had decided in consultation with Dr. Dance to make an opening in a somewhat similar case. An extensive exploration had been made, but no clot had been discovered, and at the autopsy the condition had been found to be one of embolism. Nevertheless he felt convinced that operation was justifiable in cases which were losing ground.

